# Retraction: Bioassays guided isolation of berberine from *Berberis lycium* and its neuroprotective role in aluminium chloride induced rat model of Alzheimer’s disease combined with insilico molecular docking

**DOI:** 10.1371/journal.pone.0349363

**Published:** 2026-05-14

**Authors:** The PLOS One Editors

After this article [1] was published, the following concerns were noted:

- There is a high degree of similarity between Normal control, Alzheimer’s Control, and Rivastigmine results reported in Tables 5 and 6 of [1] and Tables 1 and 2 of [2] published by the same author group.
- The *PLOS One* Editors have concerns about the article’s compliance with the PLOS Authorship policy.

The corresponding author stated that studies presented in [1] and [2] were conducted independently under different experimental conditions, and they provided the underlying data for these tables. These data were assessed by a statistics specialist, who raised concerns about the low possibility of observing this extent of numerical agreements between two separate sets of data.

In light of the unresolved concerns which question the integrity of the data, the *PLOS One* Editors retract this article.

HI and MZB did not agree with the retraction. DK, DW, MUI, ED, IuH, SA, MA, and SS either did not respond directly or could not be reached.
